# Henoch-Schönlein nephritis associated with streptococcal infection and persistent hypocomplementemia: a case report

**DOI:** 10.1186/1752-1947-4-50

**Published:** 2010-02-11

**Authors:** Francisco Rivera, Sara Anaya, Javier Pérez-Álvarez, Maria D Sánchez de la Nieta, María C Vozmediano, Julia Blanco

**Affiliations:** 1 Sección de Nefrología. Hospital General de Ciudad Real. c/Tomelloso s/n, 13005 Ciudad Real. Spain; 2 Servicio de Anatomía Patológica. Hospital Clínico Universitario San Carlos. Av. Prof. Martin Lagos, s/n. 28040 Madrid. Spain

## Abstract

**Introduction:**

Henoch-Schönlein purpura is a systemic disease with frequent renal involvement, characterized by IgA mesangial deposits. Streptococcal infection can induce an abnormal IgA immune response like Henoch-Schönlein purpura, quite similar to typical acute post-infectious glomerulonephritis. Indeed, hypocomplementemia that is typical of acute glomerulonephritis has also been described in Henoch-Schönlein purpura.

**Case presentation:**

We describe a 14-year-old Caucasian Spanish girl who developed urinary abnormalities and cutaneous purpura after streptococcal infection. Renal biopsy showed typical findings from Henoch-Schönlein purpura nephritis. In addition, she had low serum levels of complement (C4 fraction) that persisted during follow-up, in spite of her clinical evolution. She responded to treatment with enalapril and steroids.

**Conclusion:**

The case described has, at least, three points of interest in Henoch-Schönlein purpura: 1) Initial presentation was preceded by streptococcal infection; 2) There was a persistence of low serum levels of complement; and 3) There was response to steroids and angiotensin-converting enzyme inhibitor in the presence of nephrotic syndrome. There are not many cases described in the literature with these characteristics. We conclude that Henoch-Schönlein purpura could appear after streptococcal infection in patients with abnormal complement levels, and that steroids and angiotensin-converting enzyme inhibitor could be successful treatment for the disease.

## Introduction

Henoch-Schönlein purpura (HSP) is a systemic disease with frequent renal involvement, characterized by IgA mesangial deposits. Its etiology is unknown, but several infections have been described as trigger agents [1]. Streptococcal infection could induce an abnormal IgA immune responses like HSP, quite similar to typical acute post-infectious glomerulonephritis (AGN) [2,3]. Indeed, hypocomplemetemia that is typical of AGN has been also described in HSP [4].

We describe a young girl patient who developed urinary abnormalities and cutaneous purpura after streptococcal infection. Renal biopsy showed findings typical of HSP nephritis, with prominent mesangial IgA deposits. In addition, she had low serum levels of C4 that persist during follow-up, in spite of her clinical evolution. We conclude that HSP can appear after streptococcal infection in patients with abnormal complement levels.

## Case presentation

A 14-year-old Caucasian Spanish girl without previous diseases or known renal diseases, had an upper respiratory tract infection in December 2007 with malaise, no cough, tonsilar swelling, sore throat and fever >38°C, which were treated with codeine and acetaminophen. Four weeks later, she developed arthralgias and asthenia followed by purpura on legs, arms and abdomen. There was no abdominal pain or oedema. During physical examination, blood pressure was 100/45 mmHg and she did not have oedemas; she presented palpable purpura. Urine analysis revealed microscopic haematuria, proteinuria (ratio protein/creatinine 3.4 mg/mg) and granular casts with normal renal function (serum creatinine 0.8 mg/dl). Some other laboratory findings were: haemoglobin 12.7 g/dl, white cell count 6300 μ, platelet count 226000 μl, antistreptolisin-O 465 U/ml (normal under 240), serum total proteins 6 g/dL and albumin 3.7 g/dL. Coagulation study was not altered. ANA, anti-DNA, ANCAS, antibodies anti-MBG, crioglobulins, lupus anticogalulant and anticardiolipin antibodies were negatives. IgG 969 mg/dl, IgA 150 mg/dl, IgM 93 mg/dl. C3 87 mg/dl and C4 low (13 mg/dl, normal interval 15-45).

**Figure 1:**
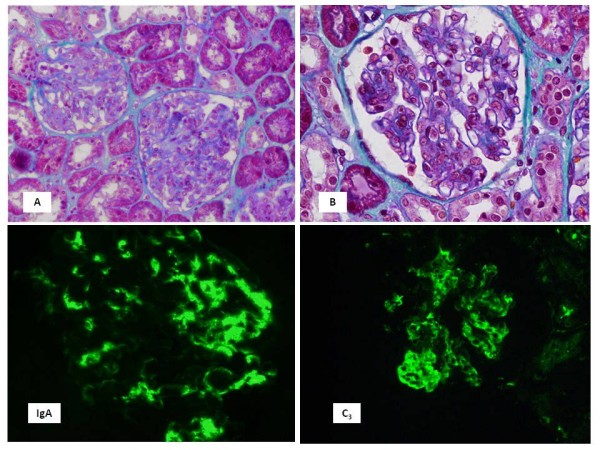
**Photomicrographs of kidney biopsy specimens**. (A and B) Endocapillary diffuse proliferation with irregular distribution among glomerular segments. (C) Mesangial deposits of IgA with some parietal deposits and (D) deposits of C3 in mesangial areas.

Abdominal ultrasound revealed normal kidneys. We performed biopsies of the purpuric lesions and the kidney. In the former, there was leukocytoclastic vasculitis. Upon renal biopsy, we examined 42 glomeruli with diffuse proliferative endocapillary proliferation with a certain degree of mesangial proliferation and increased mesangial matrix, without humps, leukocyte infiltration or crescents. Moreover, there was no vasculitis. Direct immunofluorescence revealed the deposition of granular IgA and with less intensity C3 and fibrinogen in the mesangium. The lesions were graded according to ISKD and were classified as stages II (Figure 1).

**Figure 2:**
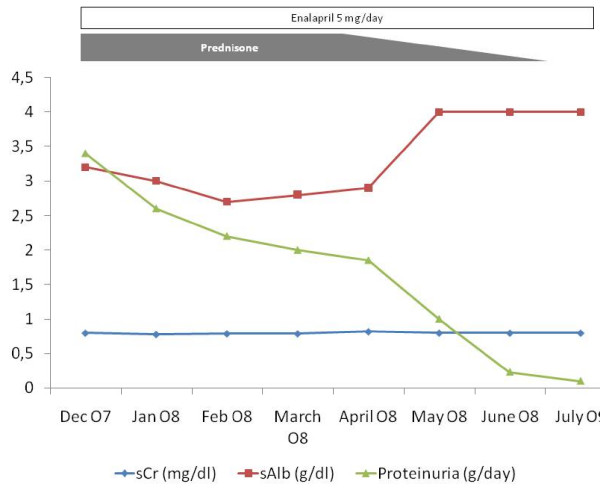
**Analytical evolution**.

**Figure 3:**
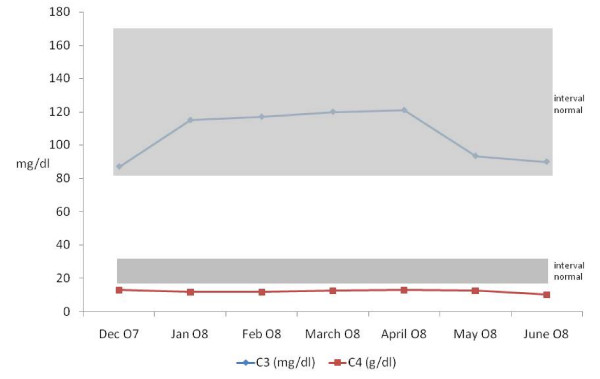
**Evolution of serum levels of complement**.

Ultrastructural study with electronic microscopy was not done. Treatment was initiated with oral prednisone 1 mg/Kg/day. Nevertheless, the illness of our patient evolved to overt nephrotic syndrome (hypoalbuminemia, oedemas) and enalapril (5 mg/day) plus aspirin (100 mg/day) were added as treatment. Prednisone was maintained for 16 weeks with progressive dose tapering. Subsequently, we observed the progressive decrease of proteinuria that remitted completely (Figure 2). In the last revision, performed nine months after initial presentation, our patient only had microhaematuria as unique manifestation of renal disease. Serum ASLO indeed decreased by more than 50% compared to initial values. Curiously, our patient maintained low levels of serum C4 without modification of serum C3 levels. See the evolution at Figure 3.

## Discussion

The case described has, at least, three points of interest in HSP: 1) Initial presentation was preceded by streptococcal infection; 2) There was persistence of low serum levels of C4; and 3) There was response to steroids and angiotensin converting enzyme inhibitor (ACEI) in the presence of nephrotic syndrome. We are going to discuss these points in the following paragraphs.

Both AGN and HSP nephritis could appear after antigen exposure with similar clinical presentation such hematuria, edemas and hypertension [2,5,6]. In this case, streptococcus infection was supported by clinical data and high serum ASLO levels that decreased subsequently. Moreover, the clinical picture and the absence of diabetes or other debilitating diseases indicates that the presence of *staphyloccocus *infection-associated glomerulonephritis mimicking IgA nephropathy seems unlikely [7]. On the one hand, the presence of hypocomplementemia would make AGN to be a more likely diagnosis. Although in this GN the complement system is usually activated by alternative pathway, it has been described as the activation by classical pathway, characterized by low levels of C4 without decrease of C3, as we observed in our patient. Moreover, GNA has also been described as having the presence of systemic vasculitis affecting skin, bowel and other organs mimicking HSP [5,6]. On the other hand, the presence of purpura and absence of typical nephritic syndrome supported the diagnosis of HSP. Indeed, it has been also described that ASLO titer positivity is associated with a significant increase in the risk of HSP and renal involvement is more common among cases with positive elevated titers [8].

Finally, renal biopsy was essential to establish definitive diagnosis, as occured in many glomerular diseases. The presence of mesangial proliferation without leukocyte infiltration and the presence of IgA deposits led us to a definitive diagnosis of HSP. These findings remark the importance of renal biopsy in the diagnosis of the majority of glomerular diseases because clinical manifestations may be similar in many different glomerular diseases [9]. We think that our patient did not have superimposed minimal change disease, although it is impossible to ensure since we did not do an electronic microscopy study. However, if the biopsy of our patient had podocyte fusion, it would explain by nephrotic proteinuria as an unspecific finding.

Although there are no serum markers of HSP, the increase of serum IgA in more than 50% of patients without modification of complement serum levels has been found [10]. However, in some patients with HSP nephritis transient hypocomplemetemia may appear [4]. Indeed, congenital defects of complement fractions are recognized predisposing factors in the development of other systemic diseases such as lupus erythematosus, Sjögren and connective tissue diseases. Furthermore, several authors have described in HSP the presence of low C4 serum levels in acute phase of nephritis in 17%, and about 20% in chronic evolution. This hypocomplemetemia is not related to the severity of the disease in most of patients [4].

In our case, the low C4 levels did not have any relation with the severity of renal evolution. Whether the hypocomplementemia is the result of complement activation after immunological activation from immune complex or indicates a congenital defect is difficult to clarify. In our case, the presence of low serum levels of C4, irrespective of clinical evolution, allows us to consider a congenital deficit because when nephropathy reached complete remission, the levels of serum C4 remained low.

Recently, it has been described that C4 null alleles were significantly more common among HSP patients than in controls and so children with C4 deficiencies may have increased risk of developing HSP [11]. Furthermore, the C4 congenital deficit is the most frequent complement congenital deficit, which in many occasions has no clinical consequences. However, in patients with other immunological alterations such abnormal IgA_1 _O-glycosilation [12], the infection with streptococcal antigens -or other antigenic stimuli- could trigger the development of HSP nephritis, as we observed in our case.

On the other hand, the so called "Nephritis-Associated-Plasmin-Receptor" (NAPlr) which has been found in the glomeruli and in sera of many patients with AGN [13,14] has been also found in renal glomeruli in 10/33 of patients with PSH and it is likely that the deposition of NAPlr in the mesangium may have a role in the pathogenesis of HSP [15]; and this antigen may be related to the pathogenesis in some patients with SHP [16]. It is attractive to speculate about streptococcal infection being involved in both GN, with the participation of NAPlr antigen. In our case, we can speculate that streptococcal infection in a patient with abnormal IgA response and congenital complement abnormalities derives from the development of HSP nephritis.

The treatment of HSP is controversial and the use of steroids and immunosuppressive drugs must be reserved for cases with a severe form of presentation. Corticosteroids produce consistent benefits and reduce the odds of developing persistent renal disease [17]. In our case, the development of nephrotic syndrome allows us to start treatment with steroids and the evolution was quite good. In our patient, we added a low dose of enalapril as an antiproteinuric measure, despite our patient having a completely normal blood pressure because of the well demonstrated beneficial effect of ACEI in idiopathic IgA nephropathy [18]. Therefore, the use of ACEI would certainly influence its evolution.

## Conclusion

We conclude that HSP could appear after streptococcal infection in patients with abnormal complement levels and irreversible glomerular injury could be prevented if treatment with steroids were initiated early.

## Consent

Written informed consent was obtained from the parents of our patient for publication of this case report and accompanying images. A copy of the written consent is available for review by the Editor-in-Chief of this journal.

## Competing interests

The authors declare that they have no competing interests.

## Authors' contributions

F Rivera, S Anaya, MD Sánchez de la Nieta and MC Vozmediano analyzed and interpreted our patient data regarding the renal disease.

J Pérez-Alvárez and J Blanco performed the histological examination of the kidney, and were major contributors in writing the manuscript. All authors read and approved the final manuscript.
